# A Decision support system (DSS) for municipal nurses encountering health deterioration among older people

**DOI:** 10.1186/s12912-016-0184-0

**Published:** 2016-11-08

**Authors:** Annica Kihlgren, Fredrik Svensson, Conny Lövbrand, Mervyn Gifford, Annsofie Adolfsson

**Affiliations:** 1 Faculty of Medicine and Health, School of Health Örebro University, SE-701 82 Örebro, Sweden; 2 Region Örebro Counties, Örebro, Sweden; 3 Ambulance Department, Örebro University Hospital, Örebro, Sweden; 4 The Centre of Women’s Health, Faculty of Health Science, Buskerud Vestfold University College, Kongsberg, Norway

**Keywords:** Elderly, Emergency care, Decision-making, Nursing homes and referral

## Abstract

**Background:**

This study is part of a larger project called ViSam and includes testing of a decision support system developed and adapted for older people on the basis of M (R) ETTS (Rapid Emergency Triage and Treatment System). The system is designed to allow municipal nurses to determine the optimal level of care for older people whose health has deteriorated. This new system will allow more structured assessment, the patient should receive optimal care and improved data transmission to the next caregiver.

**Methods:**

This study has an explanatory approach, commencing with quantitative data collection phase followed by qualitative data arising from focus group discussions over the RNs professional experience using the Decision Support system. Focus group discussions were performed to complement the quantitative data to get a more holistic view of the decision support system.

**Results:**

Using elements of the decision support system (vital parameters for saturation, pain and affected general health) together with the nurses' decision showed that 94 % of the older persons referred to hospital were ultimately hospitalized. Nurses felt that they worked more systematically, communicated more effectively with others and felt more professional when using the decision support system.

**Conclusions:**

The results of this study showed that, with the help of a decision support system, the correct patients are sent to the Emergency Department from municipal home care. Unnecessary referrals of older patients that might lead to poorer health, decreased well-being and confusion can thus be avoided. Using the decision support system means that healthcare co-workers (nurses, ambulance/emergency department/district doctor/SOS alarm) begin to communicate more optimally. There is increased understanding leading to the risk of misinterpretation being reduced and the relationship between healthcare co-workers is improved. However, the decision support system requires more extensive testing in order to enhance the evidence base relating to the vital parameters among older people and the use of the decision support system.

## Background

In Sweden around 500,000 people are older than 80 years of age, according to Statistics Sweden [1]. In 20 years’ time, it is estimated that there will be over 800,000, and by the end of the 2040s, over one million. That is, one in twenty people will be over 80 years of age. Based on these statistics, it would appear that a change in holistic terms of treatment is needed and that the care of the older person needs to be based on quality, structure and on an equal basis regardless of where in the care chain older people are cared for [2].

Older patients often have several illnesses which can make symptoms difficult to interpret. Consequently these patients are a vulnerable group and are often diagnosed as having multiple comorbidities with various functional impairments resulting in complex needs [3]. Registered nurses (RN) in Municipal Home Care (MHC) are responsible for ensuring that older people receive good care quality in Nursing Homes (NH) and also for those living in ordinary homes with MHC [4]. The responsibility of RNs working in MHC is also to act as supervisors of other staff members. MHC staffing in Sweden is usually based on enrolled nurses and nursing assistants, with only a few RNs being involved in such care.

During normal weekdays the RN’s responsibility covers one or more wards in a NH and several NH facilities, as well as patients living in their ordinary homes within the catchment areas during evening, night and holiday shifts. Furthermore, when older people are in need of emergency care, nurses are responsible for arranging their transfer to the emergency department (ED). A physician on call at a Primary Health Care Centre (PHCC) can be consulted, and outside normal daytime hours the RNs can speak with a physician on call [5]. Olsson and Hansagi [6] observed that many older people made repeated visits to the EDs and although comprising 4 % of total number of ED patients, they accounted for 18 % of the total visits. Studies from the US have reported similar findings [7, 8]. A report from the Swedish National Study on Aging and Care (SNAC) project showed that over a period of 1 year, older people who lived in NHs had about 0.5 episodes of hospital care per resident [9]. Kihlgren et al. [10] showed that one third of all NH patients were referred to an ED during 1 year. In a study by Kirsebom et al. [11] there was a total of 594 referrals to ED from a total of 431 residents (M = 1.37 each) over a 9 month period. In total, 63 % of referrals resulted in hospitalization (M = 7.12 days). NH transfer rate differed between 0.00 and 1.03 transfers per bed and was higher for the private for-profit providers than for public/private non-profit providers.

When comparing NH patients with older patients cared for at home Condelius et al. [3, 12] found a lower utilization of hospital care among NH patients. According to the Swedish Council on Technology Assessment, it is important that the initial judgment should be correct so that if and when the patient arrives at the ED, the waiting time to be seen by a doctor and the total waiting time in the ED can be reduced [6, 13].

The responsibility in Sweden for older people whose health deteriorates is shared between the county councils and the municipalities, and between different professional groups. The decision making process is complex when the older person’s health deteriorates and a decision has to be taken about a transfer to the ED. This can also be considered in a humanistic perspective, in that unnecessary referrals of older patients can lead to poorer health, decreased well-being and confusion [14–16]. Shanley [16] also showed that there are examples of patients’ being transferred to the hospital at the end of life stage, when it might have been more appropriate to provide palliative care during these final stages of life in a NH. Thus a major challenge facing RNs in MHC is to distinguish between situations where patients should be assessed as suitable for care at home or requiring transfer to the ED. "Knowing the patient" is of great importance in the care and it is a necessary condition for providing and planning high quality of care, suggesting that decision-making should be based on sound knowledge that otherwise may affect patient safety [17–19].

There are a number of studies in the literature on the frequency of older people’s visits to the ED, but there is a lack of studies focusing on RNs’ role in these referrals. There is also a lack of knowledge of tools for decision-making when an older person’s health deteriorates at home or in the NHs. However it can be seen in the international literature that several tools are used for assessment in the EDs: The Manchester Triage System [20], used in the UK and Europe. Inter-rater reliability is “moderate” to “substantial” and test-retest reliability is high. The reliability of the MTS is not influenced by nurses’ work experience. The Canadian Triage Acuity scale (CTAS) used in Canada the overall pooled coefficient for the CTAS was good K = 0.672 (CI 95 %: 0.599–0.735) [20]. For the Emergency Severity Index (ESI) used in the USA [21], the overall reliability is very good (0,79–0,9) for adult and pediatric cases and for case scenarios and live cases The five graded triage scales [22] (CTAS, ATS, MTS, METTS, ESI) are supported by some evidence for study quality and scientific evidence, [20, 23–27].

The aim of triage scales are to minimise the waiting time of patients according to the severity of their medical condition, in order to treat the most intense symptoms as quickly as possible and to reduce the negative impact on the prognosis of a prolonged delay before treatment. ED triage is a relatively modern phenomenon which was introduced in the 1950s in the United States [28]. Triage is a complex decision-making process, and several triage scales have been designed as decision-support systems [29] to guide the triage nurse to a correct decision. Apart from emergency care, triage may be used in other clinical activities, e.g. deciding on a certain investigation [30] or treatment [31, 32], but there is a need for developing these scales with a natural aging population.

Several tools for decision making for nurses are currently under development in Sweden, although knowledge is lacking in how these systems can be developed for use in the care of older people. Triage is used in acute care settings but there is a need for further development in procedures to be used as a common assessment of the older patients [33]. Within several counties in Sweden, a Rapid Emergency Triage and Treatment System (M(R)ETTS) [34, 35] is used at the EDs. This process-based triage system classifies the patients based on the severity of basic life functions, so-called vital parameters (VP) which include blood pressure, respiratory rate, temperature and oxygen saturation (SpO2), and history and underlying diseases. Whilst the VPs are already modified in some part for use in assessing older people’s health status there is nevertheless a need for their further development [31, 32]. It has been shown in previous studies that traditional VPs such as blood pressure, pulse, respiratory rate and temperature are objective measures for monitoring the patient's status with regard to acute changes, and in the assessment in chronic disease. Many physiological and pathological changes can occur with increasing age and these may affect the VPs. These changes tend to reduce the ability of the individual to adapt to physiological stress factors, especially in the frail older person [33].

Salvi et al. [36] showed in their review that the most common triage systems are not particularly suitable for older people and this supports the necessity for the further development of decision support systems which are based on multiple parameters relevant for this group.

The primary objective is to describe the normal value of vital parameters among older people receiving municipal care.

The secondary objective was to describe the outcomes when using a newly developed decision support system for decision-making when older persons’ health deteriorates and a decision is needed about the optimal level of care.

## Methods

### Study design

An explanatory approach was taken in this study, commencing with a quantitative data collection phase followed by the collection of qualitative data arising from focus group discussions (FGD) over the RNs professional experience using the Decision Support system (DSS). Quantitative data collection was used in order to provide complementary knowledge about Vital parameters (VP) and the outcomes when using DSS. Ethical approval was granted by the Uppsala Regional Ethical Review Board (registration number 2013/523).

### Context and procedure

This study is part of a larger project called ViSam and it includes the first testing of a decision support system (DSS) which was developed and adapted for older people on the basis of M (R) ETTS (Rapid Emergency Triage and Treatment System) [34, 35], hereafter called RETTS. The ViSam project is a result of consultations between the Government and the Swedish Association of Local Authorities and Regions. In 2010 an agreement was reached on structured efforts to develop health and social care for the most ill elderly people (S2010 / 1130 / ST). Örebro County region was given the responsibility for developing a model for coherent care [2].

The overall aims of the project were to develop a care plan, an information transmission system, and to introduce DSS for municipal and ambulance RNs, in order to secure the transfer of information between healthcare providers and to coordinate residents' individual needs. So far, ViSam has, on behalf of the municipalities in Örebro County and Örebro County Council, proposed an interactive model of care planning and information exchange to give nurses a tool which amalgamates safe discharge, coordinated individualized care plans and decision support. The model involves the transfer of information throughout the various processes in the health care chain, which should help ensure that individuals’ needs and prerequisites are in focus. The DSS is a tool for municipal nurses to use when the individual's health suddenly deteriorates. If the municipal nurse is unable to be present during the decision-making process then it would be possible for the ambulance nurse to perform his or her assessment by using the same DSS [2].

### Development of the Decision Support system

Various health care providers where involved in the development of DSS: RNs and leaders in municipal care from several municipalities, representatives from Primary Health Care Centre (PHCC), Ambulance, and Emergency Departments (ED) and a medical experts group (MEG). The MEG included specialist physicians from a University Hospital and from two PHCC.

The DSS was formulated as a checklist which includes reporting of VP and symptoms along with the RN's own clinical judgment in order to determine the choice of care level. DSS differs from RETTS in that the RETTS comprises five levels for the severity grade labelled by colors. In decreasing order these are Red, Orange, Yellow, Green and Blue. The DSS consists of two levels of severity grade, red and green. With the DSS, the report to the next level of care is also more structured than before, partly due to the inclusion of the communication tool, Situation, Background, Actual and Recommendation (SBAR) commonly used in the EDs, in the DSS.

In the first step, the RN registered the patient’s ID and determined whether or not there is a current decision about palliative care with adequate prescriptions for symptom relief at home. If the answer was yes, the decision would be that the patient should stay at home and contact should be made with the PHCC physician if needed.

If the answer was no, a second step was to check the VP: A, B, C, D, E and in a third step the nurse looked for exclusion symptoms for being cared for at home. These included, for example, a urinary catheter, dizziness, breathing problems, chest pain, diabetes, fever, affected general condition and back pain.

Fourthly, the RN decided if the VP was within the reference values or if there were any exclusion symptoms. The nurses filled in the DSS and could choose between a green and a red field. If there was anything that could be included in the red field it indicated that the person should be transferred to the ED. The green field was divided into two. The nurse could choose either to document what had happened and follow up the person or to phone the PHCC physician to decide what care could be given in the patient home, or if he or she should visit the PHCC. The nurses could however request the DSS outcome if they saw some extra-ordinary needs for ED care even when the DSS outcome was green. They could also decide that the person should stay at home even if the outcome was red.

### Data collection

#### Collection of quantitative data

- Step 1. All the EDs in the county used RETTS and the evaluation began following the assessment of older people arriving at the EDs over 2 weeks. The results were analyzed in MEG and a decision was made that the DSS needed to be modified in relation to the VP for saturation and circulation among older people.
- Step 2: VP among older people (*n* = 335) receiving MHC was studied at one point in one municipality. Blood pressure, pulse, oxygen saturation and respiratory rates were checked and documented.
- Step 3: VPs were then analyzed in MEG and corrections made in the DSS. One pre-test was made at the ED in the University Hospital which strengthens the adjustment of the MEGs decision.
- Step 4: The testing of the DSS began in two municipalities. The municipal RN used the DSS where she/he met a patient whose medical health had suddenly deteriorated and when he/she considered whether the patient would be transferred to the ED, or if contact would be made with the physician at the PHC or the physician on call from the PHC for continuing the care in the older person’s home. In total 281 assessments using the DSS were made during the period and 118 people were transferred to the ED. When the DSS was conducted they were sent to the Medical Responsible Nurse in each municipality. A list was drawn up after which the results from the assessment which together with the nurse documentation was sent to the ED where the nurses then completed the list with what had happened the older persons during their visits to the hospital.

The list, without any identifying any individuals, was then sent to the project leader for ViSam who together with the research team then analyzed the data.

#### Collection of qualitative data

Oral and written information of the study was given to all nurses in one community by the managers and nine nurses agreed to participate in the FGD. The researcher contacted them after they had signed the written consent. They were divided into two focus groups. One group consisted of three and one group consisted of six municipal nurses. Three of the authors were involved in the FGDs which were conducted following the standard of FGD procedures [36]. The moderators who led the FGDs were the third author together with one of the others, who took notes and assisted in asking clarifying questions. Seven questions were raised for reflection regarding how they have experienced working with the DSS. The interviews lasted between 55 and 70 min and these were recorded and transcribed verbatim by an experienced secretary.

### Data analysis

#### Statistical analysis of quantitative data

All quantitative data were analyzed using the statistical software package SPSS 18.0 (SPSS Inc., Chicago, IL, USA). Descriptive statistics, cross tabulations, sensitivity and specificity together with positive and negative predicted values were calculated. Spearman’s Chi-square and Likelihood Ratio tests were used to assess statistical significance. Positive predictive value (PPV) is the ability of the test to correctly label people who test positive, or A/(A + B. while the negative predictive value (NPV) is the ability to correctly label people who test negative, or D/(C + D) [36].

#### Analysis of qualitative data

To analyse the qualitative data a qualitative content analysis was carried out by the first author in a number of steps. The transcripts were first read several times to acquire a general sense of the whole. Meaning units related to the same central meaning and relevant to the aim of the study were then identified. These meaning units were then condensed into a description close to the text to capture the manifest content. The underlying meaning in the condensed meaning units was interpreted to determine the latent content and was then coded [37]. The codes were then compared and checked for differences and similarities, after which they were sorted into categories and after summarising the latent content in the categories, one overall theme emerged. During the analysis all authors scrutinized and repeatedly discussed the results to ensure the trustworthiness of the data analysis and the best form of presentation. Quotations are presented to illustrate the qualitative findings, these were translated from Swedish to English by an authorized translator.

## Results

During the study period 281 persons were assessed with the DSS with a mean age of 82.8 (SD 11.7) and a median age of 85 (range 67–100) years. Sixty six females took part in the study (24 %) and 49 males (17 %). For 166 participants (59 %) no information was available on sex.

### Findings from the quantitative data

#### Decision about the most appropriate care location

In the first step the RN made an assessment of if there was a decision about palliative care with adequate prescriptions for symptom relief in the home. A total of 17 % had a decision on palliative care. In a second step the VP: A, B, C, D, E was checked, see Table 1, and in a third step the nurses looked for exclusion symptoms for being cared for at home such as: urinary catheter, dizziness, breathing problems, chest pain, diabetes, fever, affected general condition, back pain, shown in Table 2.

**Table 1:** All vital parameters and their outcomes from the decisions support system *N* = 281

Vital sign	VP	Green	Red	Missing
		*n* (%)	*n* (%)	
Open airways	A	262 (93.2)	5 (1.8)	14 (4.9)
Breathing frequency within 8–25/min	B	210 (74.7)	52 (18.5)	19 (6.8)
Saturation above or equal to 92 %	B	206 (73.3)	55 (18.5)	20 (8.2)
Heartrate	C	202 (71.9)	62 (22.1)	17 (6.0)
Blood pressure	C	242 (86.1)	18 (6.4)	21 (7.5)
Degree of consciousness	D	222 (79.0)	31 (11.0)	28 (10)
Temperature	E	221 (78.6)	24 (8.5)	36 (12.8)

**Table 2:** Exclusion symptoms for being cared at home, *N* = 281

	Yes, *n* (%)	No, *n* (%)	Missing *n (%)*
Pain/Pain relief	43 (15)	222 (79)	16 (6)
Urinary catheter	14 (5)	253 (90)	14 (5)
Dizziness	20 (7)	247 (88)	14 (5)
Breathing problems	18 (6)	249 89)	14 (5)
Chest pain	26 (9)	241 (86)	14 (5)
Diabetes	12 (4)	255 (91)	14 (5)
Fever	16 (6)	251 (89)	14 (5)
Affected general health	35 (12)	232 (83)	14 (5)
Back pain	17 (6)	250 (89)	14 (5)

Exclusion symptoms among those referred to emergency care in Table 2 show that the most common symptoms were pain/pain relief and affected general health. In the fourth step the nurses decided if the VP was within the reference values and if there were any exclusion symptoms. If there was anything that indicated classification in the red field the old person was recommended to be transferred to the ED. In fact, 45 % were red and were recommended for transfer to the ED.

The results show that older persons in this study remained at home in accordance with the triage color green of DSS (*p* = 0.000) and the decision made by nurses (*p* = 0.000). The nurses’ decision that indicated Red or Green also correlated with patient hospitalisation after being transferred to ED. Additionally, the transfer to ED and hospitalisation are significantly associated with the DSS color Red (*p* = 0.000) (See Table 3).

**Table 3:** Correlation between the RNs assessment about exclusion symptoms and outcomes from DSS (*n* = 235)

Outcomes from DSS	Yes n (%)	Red	Green	Chi-square
Patient at home*	130 (55)	6	124	0.000
Patient to hospital*	86 (37)	67	19	0.000
Patient transferred*	79 (34)	52	27	0.000
Patient to medical attention*	19 (10)	1	18	0.004
Contact with PHCC physician	120 (51)	42	78	0.096
Physician on call*	18 (6)	0	14	0.003

* indicate significance

There was a significant relationship between outcomes of the VP: A and the RN’s decisions in the findings. The number of persons who stayed at home was 149 (56 %), patients referred to the ED numbered 118 (44 %) and out of these 90 persons were hospitalized, (34 % of 267). A correlation between VP: A, and contact with the PHCC physician was observed (*p* = 0.037) in the case of the RN having assessed the old person as red, *p* = 0.014 (See Table 4).

**Table 4:** Correlation between VP:A and decided care level (*n* = 267)

Outcomes from VP:A	Yes *n* (%)	Red *n* (%)	Green *n* (%)	Chi-square
Patient at home*	149 (56)	60 (22)	89 (53)	0.014
Patient to hospital*	118 (44)	66 (25)	52 (19)	0.014
Patient hospitalized*	90 (34)	56 (21)	34 (13)	0.002
Patient to medical attention	27 (10)	12 (5)	15 (6)	0.839
Contact with PHCC physician*	140 (52)	79 (30)	61 (23)	0.037

* indicate significance

#### Transferred to hospital

The outcomes from DSS in the group were compared with the triage in RETTS at the ED and evaluated in MEG during the study to further develop the DSS. When the old person’s health was evaluated using the DSS and compared with the triage RETTS there was a significant relationship, *p* = 0.000. (See Table 5).

**Table 5:** Comparing the percentage agreement of outcome DSS and RETTS at ED (*n* = 103)

	At home assessed by DSS	
	Red *n* (%)	Green *n* (%)
At Hospital used RETTS		
Red	16 (15)	0 (0)
Orange	21 (20)	12 (12)
Yellow	21 (20)	25 (24)
Green	1 (1)	5 (5)
Blue	0 (0)	2 (2)

#### Summary of statistical analysis

A stepwise multiple regression an analysis of the variables “Nurses decision, VP- B Saturation, Affected general health and “Patient to ED” was carried out (see Table 6).

**Table 6:** Dependent variable was patient to hospital compared with the independent variables

Model A	R	R square	Adjusted R square	Std. error of the estimate	Change statistics	
					R Square Change	Sig. F Change
1	.771^a^	.595	.589	.312	.595	.000
					95 % Confidence Interval for B	
Model	R	Beta In	T	Sig.	Lower Bound	Upper Bound
Constant			1.515	.131	−.070	.536
VP-B Saturation		−.109	−2.497	.013	−.230	−.027
Nurse’s Decision		.732	16.886	.000	.669	.846
Affected General Health		.105	2.374	.018	.025	.271

^a^Patient to ED

The dependent variable for Model A was Patient to hospital and the independent variables were “Affected general health”, VP- B Saturation “Nurses decision“. Of the transferred persons VP: B shows a correlation (*p* = 0,004) or the nurses decision about ED care (*p* = 0,004), see Table 6. Of the transferred persons VP: B shows a correlation (*p* = 0.004), as does the nurses decision about ED care (*p* = 0.004).

#### Sensitivity and specificity

Sensitivity and specificity were taken into account. The positive predictive value (PPV) was 0.56 and the negative predictive value (NPV) was 0.59. The positive predictive value (PPV) is the ability of the test to correctly label people who test positive, or A/(A + B). The negative predictive value is the ability to correctly label people who test negative, or D/(C + D), (See Table 7).

**Table 7:** Sensitivity and specificity of red and green outcome in the Decision system (DSS) compared with transfer to emergency department (ED) or not

	Red	Green	
To emergency department (ED)	*n* = 66	*n* = 52	PPV 56 %
Cared for at home	*n* = 60	*n* = 89	NPV 59 %
	Sensitivity 67.7 %	Specificity 38.7 %	

### Findings from the qualitative data

From the analysis of the FGD one main theme emerged: ‘*Decision making support simplifies communication and is felt by the municipal nurses to be systematic and professional’.* Three categories underlie this theme: To work more systematically, to communicate more effectively with others and to feel professional.

#### To work more systematically

The expectations for using the DSS varied among the RNs. Some saw it as just one of several changes while others saw it as a bonus for making their work more structured. They thought it could contribute to having basic measurements of VPs in the home of the older person and which could be used as a starting point in the case of deterioration. In spite of varying expectations all respondents experienced that DSS led to their work becoming more structured. Decision-making became more structured using the tool when an older person’s condition deteriorated. The systematic procedures even had a calming effect with both the patients and their relatives when the nurse checked the VPs and was able to confirm that they were normal. Many RNs said that they now used DSS regularly even if the older person’s health had not deteriorated and in order to have updated baseline information documented in the journal.

The DSS gave a confidence and sense of security for being able to defer a decision about a different level of optimal care than was available at the person’s home. The knowledge meant that the RNs could wait and see how the person’s general condition developed. This was even more important if the older person had some form of dementia illness when the RNs often made a decision of what was best for the person in question. They were also of the opinion that the most common occurrence was that a deterioration in the patient’s general condition happened progressively, and this could be seen in the journal.

*‘I actually work in a dementia ward where they are really far advanced in their dementia. So there you kind of have, you think a step ahead before you do something, so that what you do leads to something good. And most often they get worse bit by bit’*, (FGD 2).

Clarity in decision support making had even resulted in nurses being able to delegate the use of DSS to nominated nursing assistants, such as for example, the older person’s contact person. When the RN was not present the nursing assistant could carry out the checks while the nurse asked questions and filled in the DSS form via the telephone. This allowed more precise information about the care recipient to be obtained via the nursing assistant than was previously the case. This meant that they felt that they could make better decisions which in turn would be more positive for the patients, the relatives and the nursing assistants. The nursing assistants had become more vigilant in checking the documents in place at the elderly person’s home and in ensuring that the current measurements for vital parameters were in place.

*‘Now we actually have our nursing assistants who help us with the vital parameters, a few, the contact person, who do it at least once a year’, (FGD 1).*

#### To communicate more effectively with others

The RNs felt that the contact with the SOS-alarm service had become more effective compared with before, since they could now pass on VP measurements from the DSS about how the elderly person was feeling. When ordering an ambulance it was necessary that the RN could rely upon the DSS, and in doing so it made their communications clearer and they were not challenged so much by personnel from the emergency service.

*‘When you ring and order an ambulance, when you explain things it also feels like you are treated differently. The saturation is 85, he has a breathing rate of 32, rather than saying that yes he is breathing a little fast’, (FGD 2).*

They even experienced a positive difference when they handed the patient over to the ambulance personnel. The RNs felt that ambulance transport often was discussed and seen as a question of resources. It is not always obvious that an ambulance should be dispatched when they phone, but when the need is discussed in relation to the DSS, for example, when there are deviations in the VPs, then there was less discussion about the actual need for an ambulance.

*‘The situation today is that all ambulance transport is questioned. It is not obvious that an ambulance should be sent. But in this case this is also good support. Now we have this to show, that something is wrong here and there is no more discussion’, (FGD 2).*

The RNs felt that DSS had led to an increased understanding between them and the ambulance personnel. In previous discussions the RN could have questioned why the ambulance personnel checked the VPs once again after they themselves had checked them. Such discussions no longer take place.

‘*We also have an understanding of each other. Before the community nurses thought but why are you taking the parameters again? Now that there are no discussions about it either, that’s obvious’, (FGD 2).*

The use of the DSS has also meant that in the discussions with other care personnel in trying to get an understanding of the old person’s condition the RN often referred to the DSS. This was obviously a more precise way of getting information about the care recipient than before.

*‘Even if you are just sitting at the side of the road in a car you have always got the decision making tool in the back of your mind, so to speak. It’s with you when you are putting questions to the personnel’, (FGD 1).*

#### To feel professional

There had been previous situations when nurses felt they had consciously avoided being present when the ambulance personnel had been called. Feeling challenged all the time had affected them in their professionalism. The RNs said that many of them had long work experience both within acute care and in the municipality which they felt had given them a lot of experience. After the introduction of the DSS they felt that assessments had become more precise in the eyes of the ambulance personnel, which led to a feeling of increased professionalism.

*‘Because then I felt that I didn’t want to be there when the ambulance came, because so many times they were challenging. And then I felt, no I don’t want to do this… but to then realize that you’ve done a correct assessment that feels good. The work feels more structured, it’s clearer that I do what is required of me. I don’t get challenged like I did before’, (FGD 1).*

The nurses’ professionalism was also expressed when they felt secure in their intuitive assessment of the whole situation, which they did with the help of the DSS supplementary questions. They also felt they could now make better decisions which in turn created a sense of security for all involved.

*‘You do it according to this, yes, systematically. That’s what I experienced when I began to use it for the first time, that I felt more professional and then that I did something tangible which calmed both the patient and the relatives’, (FGD 2).*

It was no longer just a feeling that they got immediate confirmation that they acted correctly in the situation there and then. Some older people had even learned to complain to the RN when they did not feel well that their VPs should be checked. It then felt more professional to be able to carry out these checks more systematically, using the DSS. This means even better cooperation between the nurses and the older person.

*‘It feels very professional, this we are working with, and our patients have learned that this has to be done if they don’t feel well. It is very good’, (FGD 2).*

The nurses worked systematically with help of DSS and this meant that they felt that they could trust their assessment when debriefing their colleagues. Their responsibilities became clearer, they were not challenged and they felt more professional. The nurses expressed pride about their work with the elderly people in community care for whom they have a strong commitment.

The RNs felt, however, that difficulties could occur in using the DSS at every visit because of staff shortages. They did not think it was difficult to use the DSS by themselves. The problem lay in the fact that there were too few nurses in service, especially at nights and weekends.

*‘In such cases the difficulties are the same as we saw before – there are too few of us at nights and weekends, also daytime and evenings, that we don’t always manage to use it. We can’t be at several places at the same time’, (FGD 1).*

## Discussion

Several tools are being developed in Sweden for simplifying decision-making for nurses, but knowledge is lacking on how these tools can be adapted for use in municipal health care for older people. The main purpose of the ViSam project was to develop a care plan, an information transfer system and to introduce decision making support to municipal nurses in order to ensure information transfer between caregivers according to the care recipients’ individual needs. The model requires that the information transfer in the various processes occurs together and ensures that the individual’s needs and circumstances are in focus. The decision making tool is for nurses, among others, to use when an individual’s health status quickly deteriorates. The DSS gives the nurse an indication of where optimal care can be continued, the outcome RED means that the patient should be transferred to the ED and the outcome GREEN means that the patient can stay at home [2].

The outcomes of the VPs are distributed across all seven VPs (see Table 2), which means that all of the VPs have importance for the final outcome. It is possible to question the value of the state ‘airway is free’ when judging older people in municipal home care. However it is included because the DSS is built on the assessment concept (A-E) **A**irway, **B**reathing, **C**irculation, **D**isability, **E**xpose and Triage from RETTS, [36].

This study shows a clear connection in that all components in DSS have a meaning (see Table 7). We can also see that the different parts have relevance following the order indicated in the DSS (Table 2): breathing frequency, saturation and heart rate. Table 3 shows the exclusion symptoms where pain relief and affected general health are most common. The nurses’ own assessments and the PHCC physicians’ assessment by telephone contact are significant. The likelihood that the person assessed as GREEN can be cared for at home is high (Table 5 *p* = 0.000) and that patients with the outcome RED will be transferred to ED is also very high (Table 5, *p* = 0,000), and hospitalized (Table 6, *p* = 0.002). The results show that 94 % of those that arrived to the ED assess as RED were hospitalized unlike other studies showing 63 % resulting in hospitalization [36].

The qualitative results show that after the introduction of the DSS the nurses felt that their assessments became more precise when reporting the VP, in the eyes of the ambulance personnel and that this led to a feeling of increased professionalism. They could communicate better with the ambulance staff or the PHCC physicians when using SBAR, which can be seen in Table 5 (*p* = 0.003). Together with their clinical experience and with a holistic assessment in the third part of the DSS, the decision was better. They used their experience and knowledge and their intuition to optimize the decision [36]. This way of making decisions will reduce the probability of the nurse missing a VP that is failing. The fact that in the third step they could look for exclusion symptoms for being cared for at home will further strengthen the quality of their decision. Table 3 shows that the most common exclusion symptoms for being cared for at home were pain/pain relief 43 % and affected general health 35 %. These findings are in line with other studies, particularly in relation to affected general health [36].

Increased collaboration between all partners increases the need for information sharing, decision making and action by the various partners to be dealt with in a satisfactory manner. Studies show however that RNs working in the MHC sector are faced with serious challenges when they have to decide if a patient should be cared for at home or sent to ED [10, 18, 19]. To ‘know the patient’ is of great importance in the nursing process and the DSS seems to be one way to facilitate getting to know the patient. The nurses in this study felt that they worked more systematically. The systematic procedures even had a calming effect both with the patients and their relatives when the nurse checked the VPs. Many RNs said that they now used DSS regularly in order to have updated baseline information.

A limitation of this study is that it was performed in only in one county in Sweden and that only two out of ten municipalities were involved in the study. In Sweden the municipalities are responsible for the care of older persons and when their health deteriorates the responsibility lies with the County Council. The two municipalities were in the County Councils’ catchment areas and the three involved Eds served all the municipalities. Different health authorities in other counties may have different referral systems and procedures and this might mean that the uptake, use and experience of the new DSS could be somewhat different. For this reason further testing of the DSS is required. The development of the DSS is however supported by the results of tests for reliability and validity. Additionally, the DSS was developed from R-METTS, which is a validated instrument, and in the present work the recommended steps for instrument development have been followed [38]. Further studies are needed, for example the vital parameters should be tested with a larger group of older people and the DSS should be subjected to further psychometric evaluation.

## Conclusion

The results of this study can be interpreted as showing that with help of DSS the most appropriate patient arrives at the ED from municipal home care. This should lead to a reduction in unnecessary referrals of older patients which can be associated with poorer health, decreased well-being and avoidable confusion. When the DSS is used, it also enables municipal healthcare co-workers (nurses, ambulance/emergency department/district doctor/SOS alarm) to communicate more efficiently with each other. They communicate with each other more clearly, and this in turn leads to a reduction in the risk of misinterpretation and an improvement in the relationships with healthcare co-workers. DSS should be tested and validated more extensively in order to enhance its reliability and validity. Research about the use of prehospital nursing assessments is scarce and there is a lack of evidence based assessment instruments for use in municipal health care.

## Abbreviations

A: Airway
B: Breathing
C: Circulation
D: Disability
DSS: Decision support system
E: Expose
ED: Emergency Departments
MEG: Medical experts group
NPV: Negative predictive value
PHCC: Primary Health Care Centre
PPV: Positive predictive value
RETTS: Rapid Emergency Triage and Treatment System
RN: Register nurse
SBAR: Situation, background, actual judgement and recommendation
SOS alarm: *Save Our Souls*- alarm
VP: Vital parameter
